# Mortality with upper gastrointestinal bleeding and perforation: effects of time and NSAID use

**DOI:** 10.1186/1471-230X-9-41

**Published:** 2009-06-05

**Authors:** Sebastian Straube, Martin R Tramèr, R Andrew Moore, Sheena Derry, Henry J McQuay

**Affiliations:** 1 Department of Occupational and Social Medicine, University of Göttingen, Waldweg 37 B, D-37073 Göttingen, Germany; 2 Division of Anaesthesiology, Geneva University Hospitals and Medical Faculty, Geneva University, CH-1211 Geneva, Switzerland; 3 Pain Research, Nuffield Department of Anaesthetics, University of Oxford, Level 6 West Wing, John Radcliffe Hospital, Headington, Oxford, OX3 9DU, UK

## Abstract

**Background:**

Some people who suffer an upper gastrointestinal bleed or perforation die. The mortality rate was estimated at 12% in studies published before 1997, but a systematic survey of more recent data is needed. Better treatment is likely to have reduced mortality. An estimate of mortality is helpful in explaining to patients the risks of therapy, especially with NSAIDs.

**Methods:**

A systematic review of studies published before 1997, and between 1997 and 2008. Any study architecture was acceptable if it reported on cases who died from any cause of upper gastrointestinal bleed or perforation. Analyses were conducted separately for all cases, and those prescribed NSAID or aspirin.

**Results:**

Information was available for 61,067 cases (81% published since 1997) of whom 5,001 died. The mortality rate in all cases fell significantly, from 11.6% (95% confidence interval, 11.0 to 12.2) in pre-1997 studies to 7.4% (7.2 to 7.6) in those published since 1997. In 5,526 patients taking NSAID or aspirin, mortality increased, from 14.7% (13.6 to 15.8) before 1997 to 20.9% (18.8 to 22.9) since 1997.

**Conclusion:**

Upper gastrointestinal bleed or perforation still carries a finite risk of death. Differences in study architecture, population characteristics, risk factors, definition of mortality, and reporting of outcomes impose limitations on interpreting effect size. Data published since 1997 suggest that mortality in patients suffering from an upper gastrointestinal bleed or perforation has fallen to 1 in 13 overall, but remains higher at about 1 in 5 in those exposed to NSAID or aspirin.

## Background

Some patients who have a gastrointestinal bleed or perforation will die [1]. Risk of mortality is probably higher in older people [2], in people with concomitant diseases, or with large ulcers in the posterior duodenal bulb or on the lesser curvature [3]. Use of NSAIDs (non-steroidal anti-inflammatory drugs) or aspirin is likely to contribute to gastrointestinal bleeding and death [4].

In the largest published systematic review to date, with data from 1966 to 1996, Tramèr and colleagues assessed the mortality risk of more than two months NSAID exposure as 12% in 11,000 cases of gastrointestinal bleed or perforation, though there was a large variation of between 6% and 16% [1]. Knowing the mortality of an event may be an important element in explaining risk to patients. Mortality estimates for gastrointestinal bleeding and cardiovascular events have been used in examining how the various risks of NSAIDs and coxib (cyclooxygenase-2 selective inhibitor) use can be explained to patients [5,6].

Descriptions of the risk of dying from a gastrointestinal bleed or perforation vary significantly. For instance, an experimental study on how patients deal with risk used the following description "*A small proportion of people may die from stomach bleeding" *(compared to risk of dying from a heart attack which was given as 1 in 10 to 1 in 5) [5], while another described the risk as 10% [6]. These are quite different presentations, which may be interpreted very differently, by professionals and by patients [7].

We wanted to examine the published literature since 1997, the date of the last systematic review on mortality from upper gastrointestinal bleeding or perforation [1]. We hypothesized that better treatment of patients with a bleed or perforation might have reduced the mortality rate to below 12%. Modification of standard care leading to changes in the baseline risk has been described in patients with myocardial infarction [8], stroke [9], and with high cholesterol [10]. We also hypothesised that mortality with NSAID use might not have fallen because guidelines concerning use of gastroprotective strategies with NSAIDs in patients with at least one gastrointestinal risk factor are not followed in 3 out of 4 patients [11].

## Methods

Guidelines for quality of reporting of meta-analyses were followed where appropriate [12]. We took data from a previous systematic review [1] of studies published up to and including 1996. To identify more recent studies (published between 1997 and October 2008), we conducted a MEDLINE (PubMed) search for full publications; the date of the last search was 6 November 2008. The search was limited to 'humans' and the search strategy involved a combination of the search terms "non-steroidal anti-inflammatory", "aspirin", "upper gastrointestinal" (or "upper gastro-intestinal" or "upper GI"), "ulcer", "bleeding", "haemorrhage", "perforation", "death", and "mortality". We also contacted experts in the field for further studies. Only a small proportion of observational studies are identified through electronic searching [13,14]. Reference lists of retrieved studies, reviews, and articles that commented on formulary policy were also searched carefully for further reports. No formal quality assessments were made.

We identified reports of any kind of clinical study published in any language that contained information about mortality with upper gastrointestinal bleeding or perforation. We sought data on the number of patients experiencing upper gastrointestinal bleeds or perforations (cases), and the number of these cases who died (deaths). We sought data on the total number of cases and deaths, and those in patients using NSAIDs. NSAIDs included aspirin when it was used as an analgesic, but not low dose aspirin used for prophylaxis of myocardial infarction or stroke. Coxibs were included in the broader definition of NSAIDs.

We calculated mortality rates as the number of deaths divided by the number of cases with 95% confidence intervals (CI). This was done for all patients independent of NSAID exposure and for NSAID users separately; we were unable to identify non-NSAID users separately from all patients, and so all patient data are "contaminated" by the inclusion of patients who were taking NSAID or aspirin. Similarly, we calculated mortality rates for all times, and for the time periods 'prior to 1997' and '1997–2008' separately. Where papers reported separate datasets for different diagnoses (gastric, duodenal, or peptic ulcer; [4] for instance), we used these separate datasets in any analysis. Definitions of mortality were taken as reported in the original reports. Differences between proportions were assessed using both the t-test and Fisher's exact test, using an interactive Internet statistical package http://www.quantitativeskills.com/sisa/index.htm. Two sided tests were used, with statistical significance set at p < 0.05.

## Results

Searches, and references from the previous systematic review [1], provided 65 studies with 244,329 patients in total, of whom 61,067 experienced an upper gastrointestinal bleed or perforation, and of whom 5,001 died (we provide all the references not included in [1] in Additional file 1). Data from after 1997 made up 81% of the cases and 74% of the deaths.

There were six randomised trials (292 cases of bleed or perforation, 5 deaths), six cohort studies (12,024 bleeds or perforations, 1404 deaths), eight case-control studies (4,487 bleeds or perforations, 258 deaths), 39 case series (39,908 bleeds or perforations, 2674 deaths), three case reports (226 bleeds or perforations, 9 deaths), one yellow card report (3,443 bleeds or perforations, 576 deaths), one audit (524 bleeds or perforations, 52 deaths), and one cross sectional study (163 bleeds or perforations, 23 deaths). In total, 77 data sets from these studies contributed data on 61,067 patients who had suffered a gastrointestinal bleed or perforation.

**Figure 1:**
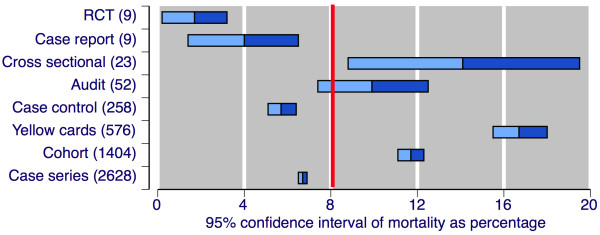
**Mortality by study type**. Mean and 95% CI of percentage mortality. Numbers in parenthesis indicate the number of deaths.

Figure 1 shows the mortality rate (as 95% CI) according to study type and number of deaths. There were substantial differences in mortality rates between different study architectures. The causal relationship between death and gastrointestinal complication was rarely examined, nor was it possible to distinguish between bleed and perforation as cause of death. Characteristics of patients who died were again rarely reported separately from the population as a whole.

Mortality was reported in different ways; as a simple report of death, 30-day mortality, death in hospital or at home, upper-gastrointestinal-related death, and others. Studies rarely stratified mortality according to a specific diagnosis, and it was not possible to perform analyses based on diagnosis. There were also inconsistencies regarding reporting of NSAID use. Few studies differentiated between use and non-use of NSAID (or aspirin). Where such a distinction was made, NSAID use was usually inferred using prescription analysis, with prescriptions for NSAID or aspirin filled within a defined period before the bleed or perforation, usually 30 days. Adherence to and details of NSAID therapy (dose, drug, frequency) were rarely specified. Because of these limitations it was feasible only to compare definite NSAID use with all patient data, whether NSAIDs or aspirin were used or not.

**Figure 2:**
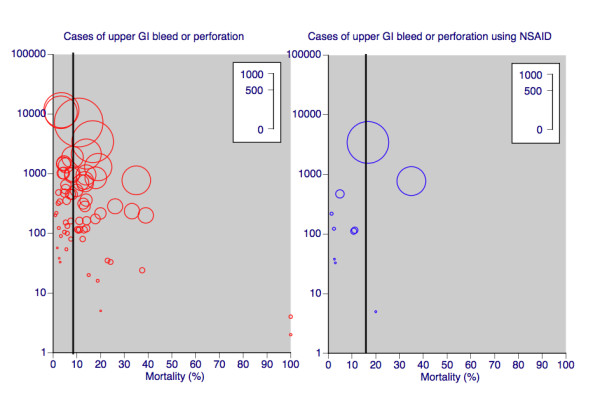
**Mortality rates in cases of upper GI (gastrointestinal) bleed or perforation, for all patients and those taking NSAID**. Size of symbol is proportional to number of deaths in each study (inset scale). Vertical line shows overall average mortality rate.

Table 1 shows mortality rates in all cases and in the 5,526 cases (9% of all cases) using NSAID or aspirin. Overall average mortality was 8.2% (95% CI 8.0 to 8.4%). In studies published before 1997 it was 11.6% (11.0 to 12.2%), and in studies published from 1997 onwards, it had fallen to 7.4% (7.2 to 7.6%). These later studies contained 81% of total cases and 74% of deaths. The mean reduction in mortality was by 4.2% (3.6 to 4.8%), a statistically significant reduction (p < 0.00001, t-test and Fisher's exact test). There was considerable variation between individual studies, particularly where the number of cases was smaller (Figure 2).

**Table 1:** Mortality rates in cases of upper gastrointestinal bleed or perforation according to date of publication and size of study, for all cases and those taking NSAID

	All Cases			Cases using NSAIDs		
	
	Number of			Number of		
				
Analysis	Cases	Deaths	Mortality (%; 95% CI)	Cases	Deaths	Mortality (%; 95% CI)
Overall	61067	5001	8.2 (8.0 to 8.4)	5526	904	16.4 (15.4 to 17.3)

**By date of publication**						
Before 1997 [1]	11361	1319	11.6 (11.0 to 12.2)	4046	595	14.7 (13.6 to 15.8)
Between 1997 and 2008	49706	3682	7.4 (7.2 to 7.6)	1480	309	20.9 (18.8 to 22.9)

**By number of cases**						
≥ 2000	35058	2410	6.9 (6.6 to 7.1)	3443	576	16.7 15.5 to 18.0)
200–1999	23147	2345	10.1 (9.7 to 10.5)	1465	297	20.3 (18.2 to 22.3)
1–199	2862	246	8.6 (7.6 to 9.6)	618	31	5.0 (3.3 to 6.7)

For cases identified as taking an NSAID or aspirin, mortality in studies published before 1997 was 14.7% (13.6 to 15.8%), rising to 20.9% (18.8 to 22.9%) in studies published from 1997 onwards. These later studies contained 27% of total NSAID cases and 34% of deaths. The mean increase in mortality was by 6.2% (3.8 to 8.5%), a statistically significant increase (p < 0.00001, t-test and Fisher's exact test).

Mortality for cases taking an NSAID was higher than for all cases, both in older and newer data sets, but excess mortality for NSAID and aspirin users was greater in more recent studies. Before 1997 mortality for NSAID and aspirin users was an average of 3.1% higher (1.9 to 4.3; p < 0.00001). Between 1997 and 2008 mortality for NSAID users was an average of 13.5% higher (11.4 to 15.6; p < 0.00001).

Most of the data (95% of cases and deaths) was in the 39 datasets with at least 200 cases, and studies with fewer than 100 cases contributed little (Table 1). Effects of study size were inconsistent. For all cases experiencing a bleed or perforation there was no consistent trend; small studies contributed 2,862 bleeds or perforations and 246 deaths. For patients on NSAIDs in these small studies, there were only 617 cases with a bleed or perforation and only 31 deaths. Here the estimated mortality rate of 5.0% was less than a third that found in larger studies (Table 1).

## Discussion

Interpretation of these results needs to be tempered by the limitations of the data. These are substantial, and include differences in study architecture, population characteristics, risk factors, definitions of mortality, reporting of outcomes according to diagnosis (perforation or ulcer bleed), and descriptions of use of NSAIDs, aspirin, or other ulcerogenic drugs. All of these are likely to impact on the magnitude of any mortality estimate, and influence any judgement on how mortality is influenced by temporal or therapeutic variables. Fifty-nine of 65 included studies were observational, where we lack any quality assessment tools that reproducibly detect bias.

For example, Figure 1 shows dramatic differences in mortality estimates between different study designs, though small numbers of actual deaths and cases imparts real uncertainty in some cases, perhaps beyond the conventional calculation of confidence intervals. Which of these designs most accurately captures the mortality rate is an interesting point. It could be argued, for instance, that randomised trials with low mortality rates reflect publication bias, or perhaps the stringent application of guidelines in inclusion criteria limits exposure of at-risk patients and that, consequently, external validity or applicability of these data may be limited. However that may be, all study designs agree that mortality with upper gastrointestinal bleeding or perforation is clinically significant.

We could see no clear reason for the different mortality rates between different study designs in which there were at least 200 deaths (case control, yellow cards, cohorts, and case series). It could be argued that the reason for this is that yellow card reporting selects more serious events, and a subsequently higher mortality rate, but that would still leave a two-fold difference of 6% to 12% mortality. Case mix probably contributes a great deal, and while meta-regression might help to quantify the extent, inconsistent reporting of patient characteristics puts limits on its power to do that. An analysis based on individual patient data would be more powerful, but it is unlikely that sufficient consistent patient data could be obtained retrospectively. These difficulties point to the need for a large ongoing prospective study capturing key demographic and diagnostic data and outcomes, an approach likely to distinguish both temporal changes and the influence of drugs, like NSAIDs, that influence gastrointestinal bleeding.

Information from 61,067 cases and 5,001 deaths was available, with more than four times as much information on mortality in patients with upper gastrointestinal bleeding or perforation published during the period between 1997 and 2008 than previously (Table 1). Results showed a significant and important reduction in mortality over time, by an average of 4%, meaning that 1 fewer person in every 20 who had an upper gastrointestinal bleed or perforation now dies, compared with before 1997. This major decrease in mortality probably reflects improved standards of care, as has been described in other settings [8-10,15]. Despite these improvements, still on average 1 in 13 cases of upper gastrointestinal bleeding or perforation dies.

It is important to stress that the figure is an average. Known risk factors for increased mortality will be increased age, comorbid conditions, and position of the bleed or perforation [2,3]. We accepted all studies reporting mortality with upper gastrointestinal bleeding, and typically these occurred in older adults in whom medicines may also increase risk of a bleed or perforation, and possibly of mortality.

At least 9% of the total cases (5,526) were prescribed an NSAID or aspirin in the period before a bleed or perforation. Not unexpectedly, mortality in this subgroup of patients was significantly higher than for all cases. NSAIDs and aspirin are known to increase the risk of upper gastrointestinal bleeding or perforation [16]. Also, NSAIDs and aspirin, when administered in analgesic doses, may delay diagnosis of, for instance, a painful perforation. Finally, NSAIDs and aspirin, through platelet inhibition, are likely to further increase the bleeding in a patient with a bleeding ulcer. It is less evident, though, why in patients with a bleed or perforation who were exposed to NSAIDs or aspirin, average mortality increased from about 15% before 1997 to about 21% after 1997. If improvement in standards of care has led to a decrease in overall mortality over time, this would imply that this improvement was not evident in cases taking NSAIDs or aspirin, which is unlikely to be the case. Overall mortality is probably a poor estimate of the non-NSAID using population because it includes patients using NSAIDs, aspirin, and sometimes both [17]. Therefore the true excess mortality in patients taking NSAIDs may be higher than that estimated.

A number of publications have pointed out that small numbers of events have the potential to produce the wrong answer because of the random play of chance [18-20]. It was possible to examine the effects of size in this analysis (Table 1). Two observations are helpful. Firstly, limiting studies with rare events to at least a study size of 200 cases, in this data set, would have meant that 95% of the information was captured in half the studies, and would not have altered the results. Secondly, relying on analyses with small numbers can produce an aberrant result, as was seen in the analysis of mortality in patients with bleed or perforation taking NSAIDs or aspirin. In the smaller studies with fewer than 200 cases each, and with a total of 31 deaths only, the event rate was less than a third that seen in larger studies with many more cases. For all cases, even smaller studies amassed almost 250 deaths, and the event rate was consistent with larger studies. This reiterates previous observation and theory [18-20] that with a small number of events in individual studies or in systematic reviews or meta-analyses, incorrect results may occur by the random play of chance.

Despite the limitations this study is important because of the large number of patients and events, with most information coming from more recently published studies. Even an average mortality rate is helpful in explaining the implications of therapy to individual patients, as well in calculating the economic implications of prescribing policies.

The limitations of the studies included in this review highlight how better investigational criteria might be applied in future to better understand and more reliably quantify the relationship between a gastrointestinal bleed or perforation and death. Only very large prospective studies that detail diagnosis, medication, comorbid conditions, and report the details separately for those with the event and those who die are likely to be useful, as a nationwide study from Spain exemplifies [4].

## Conclusion

Upper gastrointestinal bleed or perforation still carries a finite risk of death. Differences in study architecture, population characteristics, risk factors, definition of mortality, and reporting of outcomes impose limitations on interpreting effect size. Data published since 1997 suggest that overall mortality in any patient with a bleed or perforation has fallen over time but is still about 1 in 13. Not unexpectedly, mortality is even higher in patients with a bleed or perforation who are exposed to NSAID or aspirin. New knowledge is that in these patients, mortality appears to have increased over time to about 1 in 5 since 1997. Reasons for this increase remain to be elucidated.

## Competing interests

RAM, HJM, and MRT have received research grants, consulting, or lecture fees from pharmaceutical companies. SS, RAM, HJM, SD, and MRT have also received research support from charities or government sources at various times. RAM is the guarantor. No author has any direct stock holding in any pharmaceutical company.

## Authors' contributions

RAM and SS were involved with the original concept, planning the study, searching, writing it, analysis, and preparing a manuscript. MRT provided information from the original review. SS and RAM performed calculations and analysis. SD, MRT and HJM were involved with planning, and writing. All authors read and approved the final manuscript.

## Editor's note

The full pre-publication history of this manuscript is not currently available online. If you would like to see the original manuscript submission and the original peer review reports, please contact editorial@biomedcentral.com.

## Pre-publication history

The pre-publication history for this paper can be accessed here:

http://www.biomedcentral.com/1471-230X/9/41/prepub

## Supplementary Material

Additional file 1 References for all papers included in this review that were published from 1997 onwards. Please see [1] for all papers published before 1997. Click here for file
