# Supplementary material for: Mortality with upper gastrointestinal bleeding and perforation: effects of time and NSAID use
Source: BMC Gastroenterol. 2009 Jun 5;9:41. doi: 10.1186/1471-230X-9-41 (PMC2698873; doi:10.1186/1471-230X-9-41)
Supplement: Additional file 1 — References for all papers included in this review that were published from 1997 onwards. Please see [1] for all papers published before 1997. [file 1471-230X-9-41-S1.doc]

**Appendix**

Here we list references for all papers included in this review that were published from 1997 onwards. Please see [1] for all papers published before 1997.

1. Archimandritis A, Tsirantonaki M, Tryphonos M, Kourtesas D, Sougioultzis S, Papageorgiou A, Tzivras M: **Ranitidine versus ranitidine plus octreotide in the treatment of acute non-variceal upper gastroitestinal bleeding: a prospective randomised study.** *Curr Med Res Opin* 2000, **16**:178-183.
2. Blatchford O, Davidson LA, Murray WR, Blatchford M, Pell J: **Acute upper gastrointestinal haemorrhage in west of Scotland: case ascertainment study.** *BMJ* 1997, **315**:510-514.
3. Blot WJ, Fischer T, Nielsen GL, Friis S, Mumma M, Lipworth L, DuBois R, McLaughlin JK, Sorensen HAT: **Outcome of upper gastro-intestinal bleeding and use of ibuprofen versus paracetamol.** *Pharm World Sci* 2004, 26:319-323.
4. Chen CC, Chong CF, Kuo CD, Wang TL: **Silent myocardial ischemia in coronary artery disease patients under aspirin therapy presenting with upper gastrointestinal hemorrhage.** *J Gastroenterol Hepatol* 2007, 22:13-17.
5. Czernichow P, Hochain P, Nousbaum JB, Raymond JM, Rudelli A, Dupas JL, Amouretti M, Gouerou H, Capron MH, Herman H, Colin R: **Epidemiology and course of acute upper gastro-intestinal haemorrhage in four French geographical areas.** *Eur J Gastroenterol Hepatol* 2000, **12**:175-181.
6. Di Fiore F, Lecleire S, Merle V, Herve S, Duhamel C, Dupas JL, Vandewalle A, Bental A, Gouerou H, Le Page M, Amouretti M, Czernichow P, Lerebours E: **Changes in characteristics and outcome of acute upper gastrointestinal haemorrhage: a comparison of epidemiology and practices between 1996 and 2000 in a multicentre French study.** *Eur J Gastroenterol Hepatol* 2005, **17**:641-647.
7. Fisher L, Fisher A, Pavli P, Davis M**: Perioperative acute upper gastrointestinal haemorrhage in older patients with hip fracture: incidence, risk factors and prevention.** *Aliment Pharmacol Ther* 2007, **25**:297-308.
8. Gallerani M, Simonato M, Manfredini R, Volpato S, Vigna GB, Fellin R; Investigators of the GIFA Study (Gruppo Italiano di Farmacovigilanza nell'Anziano): **Risk of hospitalization for upper gastrointestinal tract bleeding.** *J Clin Epidemiol* 2004, **57**:103-110.
9. Garcia Rodriguez LA, Cattaruzzi C, Troncon MG, Agostinis L: **Risk of hospitalization for upper gastrointestinal tract bleeding associated with ketorolac, other nonsteroidal anti-inflammatory drugs, calcium antagonists, and other antihypertensive drugs.** *Arch Intern Med* 1998, **158**:33-39.
10. Geyer M, Stamenic I, Buhler H, Bertschinger P: **[Epidemiology of gastrointestinal bleeding in the elderly]** *Schweiz Rundsch Med Prax*, 2006 **95**:757-765.
11. Gutthann SP, Garcia Rodriguez LA, Raiford DS: **Individual nonsteroidal antiinflammatory drugs and other risk factors for upper gastrointestinal bleeding and perforation.** *Epidemiology* 1997, 8:18-24.
12. Jayaprakash A, McGrath C, McCullagh E, Smith F, Angelini G, Probert C: **Upper gastrointestinal haemorrhage following cardiac surgery: a comparative study with vascular surgery patients from a single centre.** *Eur J Gastroenterol Hepatol* 2004 **16**:191-194.
13. Kapur KC, Green JT, Turner RG, Swift J, Srivastava ED, Allison MC: **Auditing mortality from upper gastrointestinal haemorrhage: impact of a high dependency unit.** *J R Coll Physicians Lond* 1998, **32**:246-250.
14. Klebl FH, Bregenzer N, Schofer L, Tamme W, Langgartner J, Scholmerich J, Messmann H: **Comparison of inpatient and outpatient upper gastrointestinal haemorrhage.** *Int J Colorectal Dis* 2005, **20**:368-375.
15. Lanas A, Perez-Aisa MA, Feu F, Ponce J, Saperas E, Santolaria S, Rodrigo L, Balanzo J, Bajador E, Almela P, Navarro JM, Carballo F, Castro M, Quintero E; Investigators of the Asociacion Espanola de Gastroenterologia (AEG): **A nationwide study of mortality associated with hospital admission due to severe gastrointestinal events and those associated with nonsteroidal antiinflammatory drug use.** *Am J Gastroenterol* 2005, **100**:1685-1693.
16. Lim CH, Heatley RV: **Prospective study of acute gastrointestinal bleeding attributable to anti-inflammatory drug ingestion in the Yorkshire region of the United Kingdom.** *Postgrad Med J* 2005, **81**:252-254.
17. Marmo R, Koch M, Cipolletta L, Capurso L, Pera A, Bianco MA, Rocca R, Dezi A, Fasoli R, Brunati S, Lorenzini I, Germani U, Di Matteo G, Giorgio P, Imperiali G, Minoli G, Barberani F, Boschetto S, Martorano M, Gatto G, Amuso M, Pastorelli A, Torre ES, Triossi O, Buzzi A, Cestari R, Della Casa D, Proietti M, Tanzilli A, Aragona G, Giangregorio F, Allegretta L, Tronci S, Michetti P, Romagnoli P, Nucci A, Rogai F, Piubello W, Tebaldi M, Bonfante F, Casadei A, Cortini C, Chiozzini G, Girardi L, Leoci C, Bagnalasta G, Segato S, Chianese G, Salvagnini M, Rotondano G: **Predictive factors of mortality from nonvariceal upper gastrointestinal hemorrhage: a multicenter study**. *Am J Gastroenterol* 2008 **103**:1639-1647.
18. Ng W, Wong WM, Chen WH, Tse HF, Lee PY, Lai KC, Li SW, Ng M, Lam KF, Cheng X, Lau CP: **Incidence and predictors of upper gastrointestinal bleeding in patients receiving low-dose aspirin for secondary prevention of cardiovascular events in patients with coronary artery disease.** *World J Gastroenterol* 2006, **12**:2923-2927.
19. Nousbaum JB, Hochain P, Kerjean A, Rudelli A, Lalaude O, Herman H, Czernichow P, Dupas JL, Amouretti M, Gouerou H, Colin R: **[Hemorrhaging eso-gastro-duodenal ulcers: epidemiology and management. A multicenter prospective study]** *Ann Chir* 1999, **53**:942-948.
20. Paspatis GA, Matrella E, Kapsoritakis A, Leontithis C, Papanikolaou N, Chlouverakis GJ, Kouroumalis E: **An epidemiological study of acute upper gastrointestinal bleeding in Crete, Greece.** *Eur J Gastroenterol Hepatol* 2000, **12**:1215-1220.
21. Peura DA, Lanza FL, Gostout CJ, Foutch PG: **The American College of Gastroenterology Bleeding Registry: preliminary findings.** *Am J Gastroenterol* 1997, **92**:924-928.
22. Ramsoekh D, van Leerdam ME, Rauws EA, Tytgat GN: **Outcome of peptic ulcer bleeding, nonsteroidal anti-inflammatory drug use, and Helicobacter pylori infection.** *Clin Gastroenterol Hepatol* 2005, **3**:859-864.
23. Ruigómez A, García Rodríguez LA, Hasselgren G, Johansson S, Wallander MA: **Overall mortality among patients surviving an episode of peptic ulcer bleeding.** *J Epidemiol Community Health* 2000, **54**:130-133.
24. Schemmer P, Decker F, Dei-Anane G, Henschel V, Buhl K, Herfarth C, Riedl S: **The vital threat of an upper gastrointestinal bleeding: Risk factor analysis of 121 consecutive patients**. *World J Gastroenterol* 2006, **12**:3597-3601.
25. Thomsen RW, Riis A, Munk EM, Nørgaard M, Christensen S, Sørensen HT: **30-day mortality after peptic ulcer perforation among users of newer selective COX-2 inhibitors and traditional NSAIDs: a population-based study.** *Am J Gastroenterol* 2006, **101**:2704-2710.
26. Thomsen RW, Riis A, Christensen S, Nørgaard M, Sørensen HT: **Diabetes and 30-day mortality from peptic ulcer bleeding and perforation: a Danish population-based cohort study.** *Diabetes Care* 2006, **29**:805-810.
27. van Leerdam ME, Vreeburg EM, Rauws EA, Geraedts AA, Tijssen JG, Reitsma JB, Tytgat GN: **Acute upper GI bleeding: did anything change? Time trend analysis of incidence and outcome of acute upper GI bleeding between 1993/1994 and 2000.** *Am J Gastroenterol* 2003, **98**:1494-1499.
28. Wilcox CM, Clark WS: **Association of nonsteroidal antiinflammatory drugs with outcome in upper and lower gastrointestinal bleeding.** *Dig Dis Sci* 1997, **42**:985-989.
29. Yilmaz S, Bayan K, Dursun M, Canoruc F, Kilinc N, Tuzun Y, Danis R, Ertem M: **Does adding misoprostol to standard intravenous proton pump inhibitor protocol improve the outcome of aspirin/NSAID-induced upper gastrointestinal bleeding?: a randomized prospective study.** *Dig Dis Sci* 2007, **52**:110-118.
30. Zimmerman J, Shohat V, Tsvang E, Arnon R, Safadi R, Wengrower D: **Esophagitis is a major cause of upper gastrointestinal hemorrhage in the elderly.** *Scand J Gastroenterol* 1997, **32**:906-909.
